# Immediate cardiopulmonary responses to consecutive pulmonary embolism: a randomized, controlled, experimental study

**DOI:** 10.1186/s12890-024-03006-9

**Published:** 2024-05-14

**Authors:** Mads Dam Lyhne, Jacob Gammelgaard Schultz, Christian Schmidt Mortensen, Anders Kramer, Jens Erik Nielsen-Kudsk, Asger Andersen

**Affiliations:** 1 Department of Clinical Medicine, Aarhus University, Palle Juul Jensens Boulevard 82, Aarhus N, 8200 Denmark; 2 Department of Anaesthesiology and Intensive Care, Aarhus University Hospital, Palle Juul Jensens Boulevard 99, Aarhus N, DK-8200 Denmark; 3 Department of Cardiology, Aarhus University Hospital, Palle Juul Jensens Boulevard 99, Aarhus N, 8200 Denmark

**Keywords:** Right ventricular function, Pulmonary circulation, Right ventricular afterload, Ventilation-perfusion mismatch, Gas exchange, Animal model

## Abstract

**Background:**

Acute pulmonary embolism (PE) induces ventilation-perfusion mismatch and hypoxia and increases pulmonary pressure and right ventricular (RV) afterload, entailing potentially fatal RV failure within a short timeframe. Cardiopulmonary factors may respond differently to increased clot burden. We aimed to elucidate immediate cardiopulmonary responses during successive PE episodes in a porcine model.

**Methods:**

This was a randomized, controlled, blinded study of repeated measurements. Twelve pigs were randomly assigned to receive sham procedures or consecutive PEs every 15 min until doubling of mean pulmonary pressure. Cardiopulmonary assessments were conducted at 1, 2, 5, and 13 min after each PE using pressure-volume loops, invasive pressures, and arterial and mixed venous blood gas analyses. ANOVA and mixed-model statistical analyses were applied.

**Results:**

Pulmonary pressures increased after the initial PE administration (*p* < 0.0001), with a higher pulmonary pressure change compared to pressure change observed after the following PEs. Conversely, RV arterial elastance and pulmonary vascular resistance was not increased after the first PE, but after three PEs an increase was observed (*p* = 0.0103 and *p* = 0.0015, respectively). RV dilatation occurred following initial PEs, while RV ejection fraction declined after the third PE (*p* = 0.004). RV coupling exhibited a decreasing trend from the first PE (*p* = 0.095), despite increased mechanical work (*p* = 0.003). Ventilatory variables displayed more incremental changes with successive PEs.

**Conclusion:**

In an experimental model of consecutive PE, RV afterload elevation and dysfunction manifested after the third PE, in contrast to pulmonary pressure that increased after the first PE. Ventilatory variables exhibited a more direct association with clot burden.

**Supplementary Information:**

The online version contains supplementary material available at 10.1186/s12890-024-03006-9.

## Background

Acute pulmonary emboli (PE) induce a ventilation-perfusion mismatch through combined mechanical vascular obstruction and pulmonary vasoconstriction. This combination augments right ventricular (RV) afterload [1, 2] leading to RV dilatation and progressive RV failure in severe cases [1, 3]. Acute PE is a cardiac emergency where death can ensue within hours due to RV failure [4]. Diagnosis of PE is challenged by the urgency of the disease, the unspecific symptoms and the fact that patients may have been severely affected initially but have clinically improved by the time they seek medical advice. This change in symptoms and severity argues that acute PE is a dynamic condition that warrants evaluation of hemodynamics and RV function.

Despite the acute nature of PE, most assessments of RV function in clinical studies are delayed several hours [5, 6] or even days after symptom onset [7, 8]. Even in highly controlled pre-clinical setups, initial hemodynamic evaluations of PE are often performed 30 min or later after the event [9–11], leaving the physiological responses occurring immediately following acute PE largely unexplored. Given the rapid onset of fatal outcomes within minutes to hours and the complexities of conducting diagnostic procedures in critically ill patients, understanding the immediate cardiopulmonary responses may aid physicians diagnosing and treating patients with acute PE.

Previously, we have developed a porcine model simulating acute central PE [12, 13], allowing early and consecutive measurements with high temporal resolution. The objective of our present exploratory study is to describe the cardiopulmonary responses occurring within minutes after consecutive acute PE events. We hypothesized that successive PE episodes induced stepwise circulatory and ventilatory deteriorations.

## Methods

### Design

**Fig. 1:**
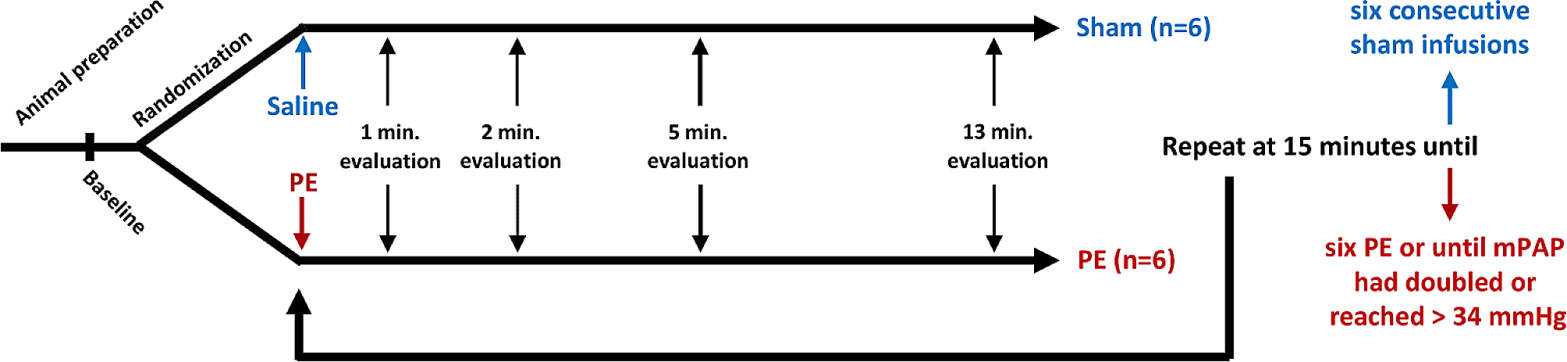
Study design

We conducted a randomized, controlled, blinded experimental study of repeated measurements. Baseline assessment was performed after stabilizing the animals. Subsequently, animals were randomly assigned in a 1:1 ratio to receive pulmonary emboli (*n* = 6) or sham procedures (*n* = 6). In the PE group, consecutive emboli (up to six emboli) were administered every 15 min until the mean pulmonary arterial pressure (mPAP) had doubled from baseline or reached a minimum mPAP > 34 mmHg 15 min following the last PE. Cardiopulmonary responses were assessed at 1, 2, 5, and 13 min after each PE episode. Load-independent biventricular pressure-volume (PV) data were recorded solely at the 5-minute timepoint. Sham animals underwent six consecutive sham-embolisations with an equivalent volume of saline, followed by evaluations. See Fig. 1.

Inclusion criteria involved a baseline mPAP < 25 and post-instrumentation sinus rhythm. Exclusion criteria comprised infection, significant hemorrhage (> 1 L) during instrumentation, or the inability to complete the protocol.

### Animals and Ethics

This is a sub-analysis of a previously published study [12] involving Danish female slaughter pigs weighing 60 ± 2 kg. The Danish Animal Research Inspectorate approved the study (no.: 2016-15-0201-00840), ensuring compliance with Danish legislation, the ARRIVE guidelines, and the 3R framework [14]. We ensured humane endpoints, as sufficient levels of anesthesia were checked regularly throughout protocol, and the animals were post-protocol euthanized with a lethal dose of pentobarbital while in deep anesthesia.

### Animal Preparation

We previously detailed the model, preparation, instrumentation, and surveillance [12]. Notably, animals were anesthetized with use of propofol (2.5 mg/kg/h, Propolipid, Fresnius Kabi, Germany) and fentanyl (6.25 mg/kg/h, Hameln Pharma, Germany). They were mechanically ventilated (Datex-Ohmeda S/5 Avance, GE Healthcare, USA) with an adjusted O_2_ inspiratory fraction to achieve arterial partial pressure of oxygen (PaO_2_) between 17 and 21 kPa. Respiratory frequency targeted an end-tidal CO_2_ (EtCO_2_) of 5.0-5.5 kPa.

### Invasive accesses and measurements

As described previously, a 26 F sheath (Dry-Seal, Gore Medical, USA) was fluoroscopically placed via the right external jugular vein for embolus administration and right heart catheterization [12]. Additionally, an IVC balloon (PTS-X, Metec, Denmark) was inserted through the femoral vein. PV catheters were advanced through the left jugular vein and carotid artery into the right and left ventricle, respectively [15].

To maintain normal physiology, we utilized a closed-chest approach. Details of the instrumentation have been previously described [12, 15]. A Swan-Ganz catheter (7.5 F, CCOmbo, Edwards Lifescience, USA) was positioned in the pulmonary trunk to measure mean pulmonary arterial pressure (mPAP). A 7 F sheath was placed in the left femoral artery for mean arterial pressure (MAP) measurements. Pulmonary and systemic vascular resistances (PVR and SVR, respectively) were calculated. Admittance-based PV catheters (emka Technologies, Paris, France, and ADV500, Transonic Scisense, London, Canada) facilitated bi-ventricular PV measurements. Data were recorded in LabChart through PowerLab 8/35 (ADInstruments, Oxford, UK). Most PV data were captured during continuous ventilation as an average over three respiratory cycles. Load-independent measurements occurred during transient apnea during IVC occlusion. PV data analysis was performed with the observer blinded to its source.

### Embolus formation

The model has been validated and described elsewhere [12, 13]. Each of six plastic tubes were filled with 30 mL of the animals’ blood forming cylindric clots mimicking thrombus from a deep leg vein. The clots were later introduced en bloc through the 26 F sheath, embedded in saline, to induce central PE [13]. 

### Blood gases

Simultaneous arterial and mixed venous blood samples were drawn for analysis of partial pressures of O_2_ and CO_2_ (PaO_2_, PaCO_2_, PvO_2_, and PvCO_2_, respectively, using ABL90 Flex Plus, Radiometer Medical, Denmark). Physiological dead space was computed following Bohr’s principle, and pulmonary shunt fraction was calculated [16]. 

### Statistics

Sample size calculations were previously described [12]. The Shapiro-Wilks test and QQ-plots were used to assess normality. Normally distributed data are presented as mean ± SD, otherwise as median [interquartile range]. Two-way ANOVA or mixed-effect analysis assessed overall differences between randomization groups. To control for power in multiple comparisons, hierarchical testing was applied from the last time point backward. Paired t-tests (non-parametric when necessary) evaluated differences in 1-minute measurements immediately preceding any PE induction. We used linear least-squares regression analysis without weighting or interaction to correlate the number of PE (clot mass) or sham inductions to cardiopulmonary variables, each forced through the baseline mean value. The slope of the regression line was compared to a hypothetical value of 0, i.e. no change in the given variable by the number of PE/sham inductions. GraphPad Prism 8.3.0 (GraphPad Software, LCC, CA) was used for statistical analyses, with a p-value < 0.05 considered statistically significant.

## Results

Twelve out of thirteen animals were included in the study. One was excluded due to equipment failure. Baseline cardiopulmonary variables were similar among the included animals (12).

### RV afterload

**Fig. 2:**
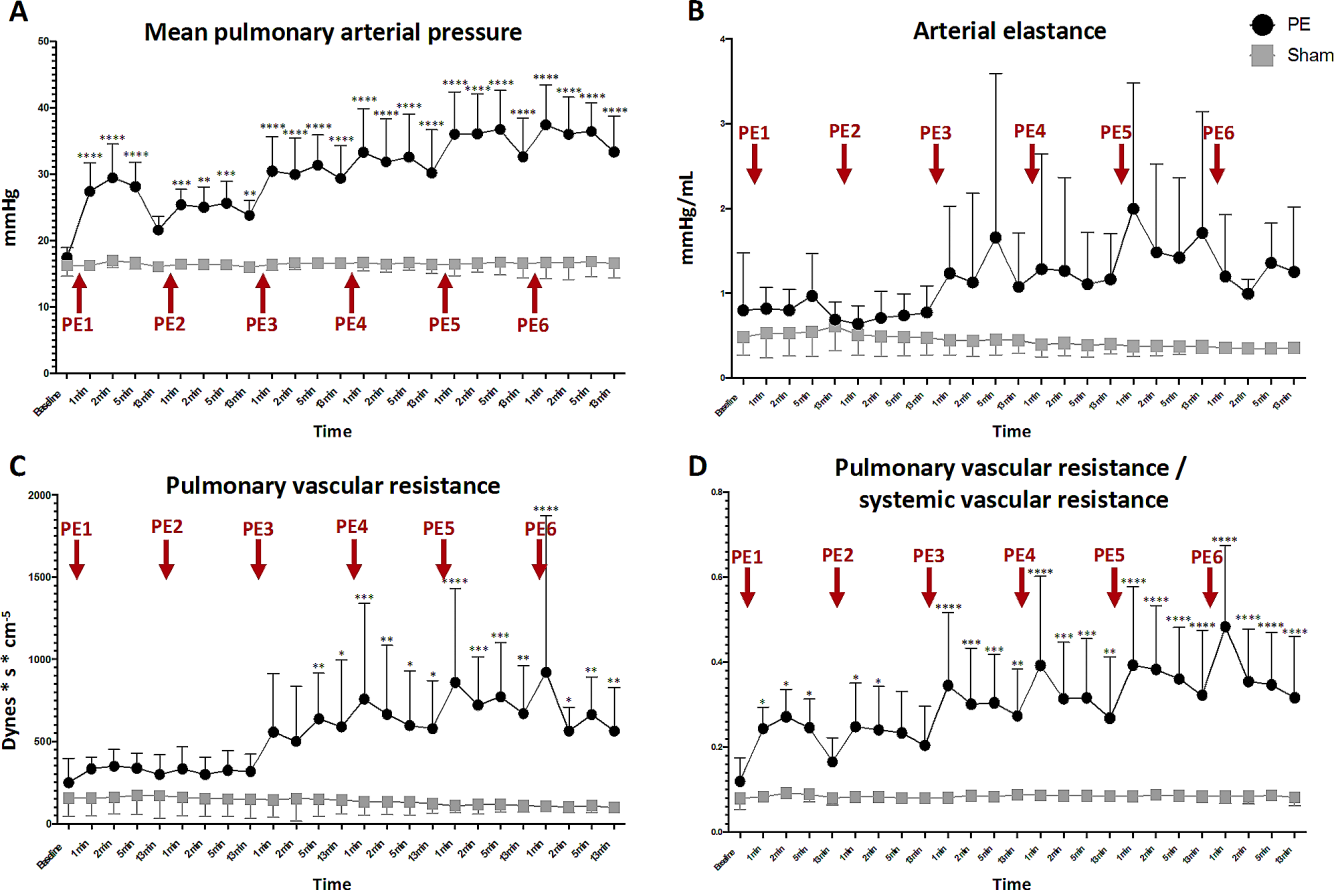
Measures of right ventricular afterload Responses to consecutive, acute pulmonary emboli on mean pulmonary arterial pressure (**A**), arterial elastance (**B**), pulmonary vascular resistance alone (**C**) and indexed to systemic vascular resistance (**D**). Please note the different responses to increased clot burden

Consecutive PE increased mPAP (ANOVA *p* < 0.0001, Fig. 2A), particularly pronounced after the first PE (PE1 9.9 ± 5.5 vs. PE2 3.9 ± 1.5 mmHg, *p* = 0.025). Pulmonary vascular resistance and pulmonary arterial elastance (Ea) increased progressively (ANOVA *p* = 0.0015 and *p* = 0.0103, respectively) but from a later time point after the third PE (Fig. 2B-C). The PVR/SVR ratio increased notably after the first PE (ANOVA *p* = 0.0005, Fig. 2D).

### RV function

**Fig. 3:**
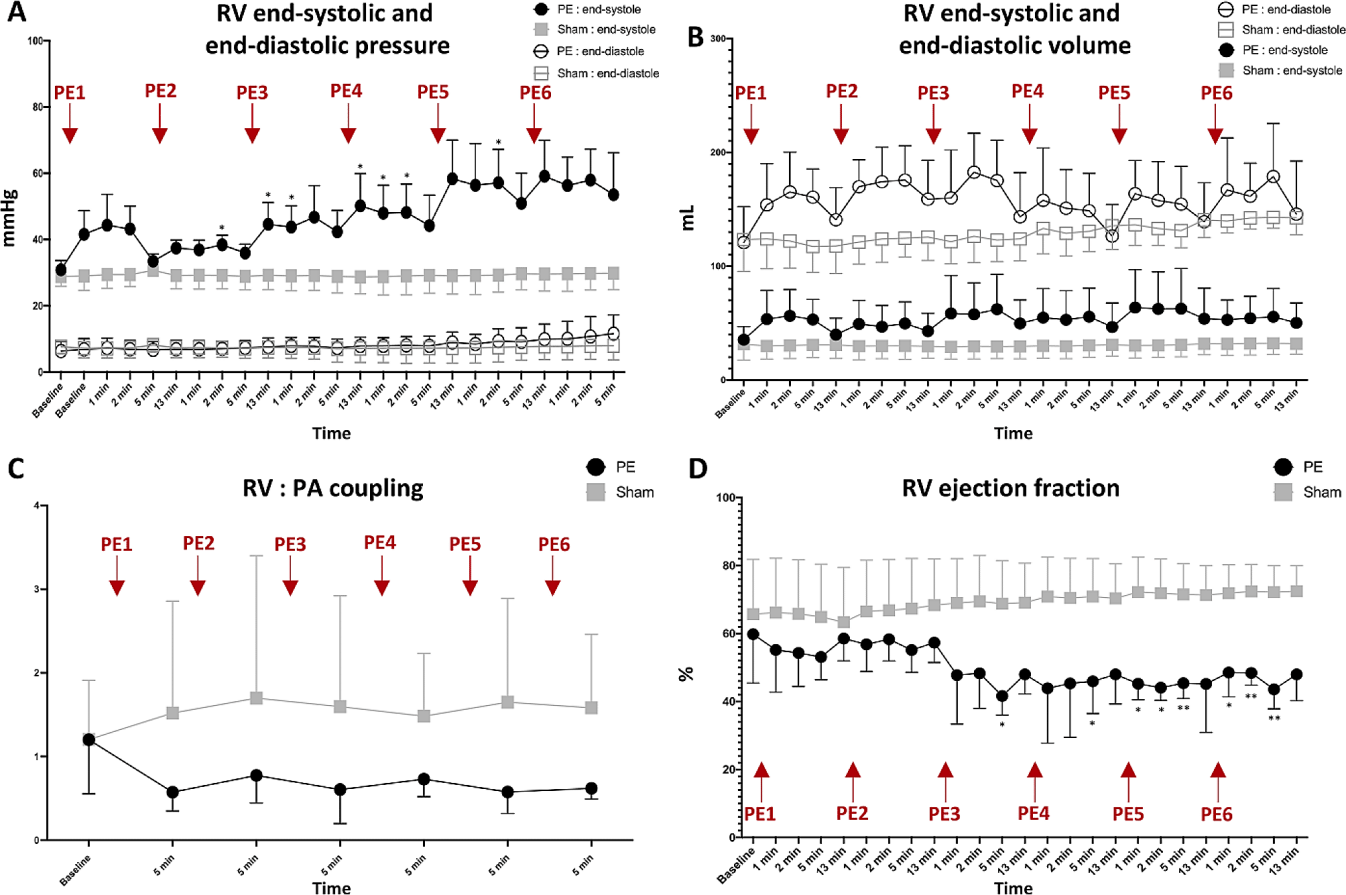
Right ventricular function in acute pulmonary embolism

RV end-systolic pressure (ESP) was increased, particularly after the first PE (ANOVA *p* < 0.0001, Fig. 3A). RV end-systolic volume (ESV) and RV end-diastolic volume (EDV) increased, but not consistently (Fig. 3B). RV stroke volume (SV) initially increased followed by declining RV output, although the overall ANOVA was not statistically significant (*p* = 0.109). RV ejection fraction (EF) decreased (*p* = 0.004, Fig. 3D) despite increased RV mechanical work (*p* = 0.003). End-systolic elastance (Ees) remained unchanged (*p* = 0.56), and the Ees/Ea ratio decrease was non-significant (*p* = 0.095, Fig. 3C). Acute changes in RV diastolic function were minimal (*p* = 0.723 for RV end-diastolic elastance, *p* = 0.0246 for RV dP/dt_min_).

### Clot burden correlation

In PE animals, all variables correlated with clot mass, particularly pulmonary pressure, PVR, and RV end-systolic pressure (Table 1). No correlations were observed in the sham group.

**Table 1:** Correlation between number of sham/PE inductions and cardiopulmonary variables

Variable	Group	Slope	95%CI	*p*-value vs. 0	r^2^
**Hemodynamics**					
HR, bpm	PE	2.55	0.59–4.51	0.01	0.08
	Sham	0.78	-1.69-3.25	0.52	0.002
mPAP, mmHg	PE	3.46	2.88–4.04	< 0.0001	0.61
	Sham	0.04	-0.11-0.19	0.57	0.01
PVR, dynes	PE	83.9	46.6-121.1	< 0.0001	0.25
	Sham	-6.1	-21.0-8.8	0.41	0.02
**RV function**					
RV end-systolic pressure, mmHg	PE	3.34	2.56–4.13	< 0.0001	0.51
	Sham	-0.31	-0.90-0.26	0.30	0.009
RV end-diastolic volume, mL	PE	6.62	1.19–12.06	0.02	0.05
	Sham	1.81	− 0.147 − 5.10	0.27	0.03
RV arterial elastance, mmHg/mL	PE	0.067	-0.007-0.15	0.07	0.08
	Sham	-0.014	-0.044-0.016	0.34	0.03
RV ejection fraction, %	PE	-2.96	-4.32- -1.60	0.0001	0.24
	Sham	1.08	-0.86-3.03	0.26	0.02
**Ventilatory function**					
PaO_2_, kPa	PE	-1.41	-1.87- -0.95	< 0.0001	0.27
	Sham	-0.36	-0,51- -0.18	0.0002	0.16
PaCO_2_, kPa	PE	0.33	0.25–0.41	< 0.0001	0.46
	Sham	0.10	0.07–0.13	< 0.0001	0.29
EtCO_2_, kPa	PE	-0.07	-0.12- -0.03	0.004	0.10
	Sham	0.002	-0.038-0.042	0.91	0.0004

All cardiopulmonary variables correlate with clot mass in the PE group compared to almost none in the sham group. Abbreviations: CI, confidence interval; PE, pulmonary embolism; HR, heart rate; mPAP, mean pulmonary arterial pressure; PVR, pulmonary vascular resistance; RV, right ventricular; PaO2, arterial partial pressure of oxygen; PaCO2, arterial partial pressure of carbon dioxide; EtCO2, end-tidal carbon dioxide

### Pulmonary function

**Fig. 4:**
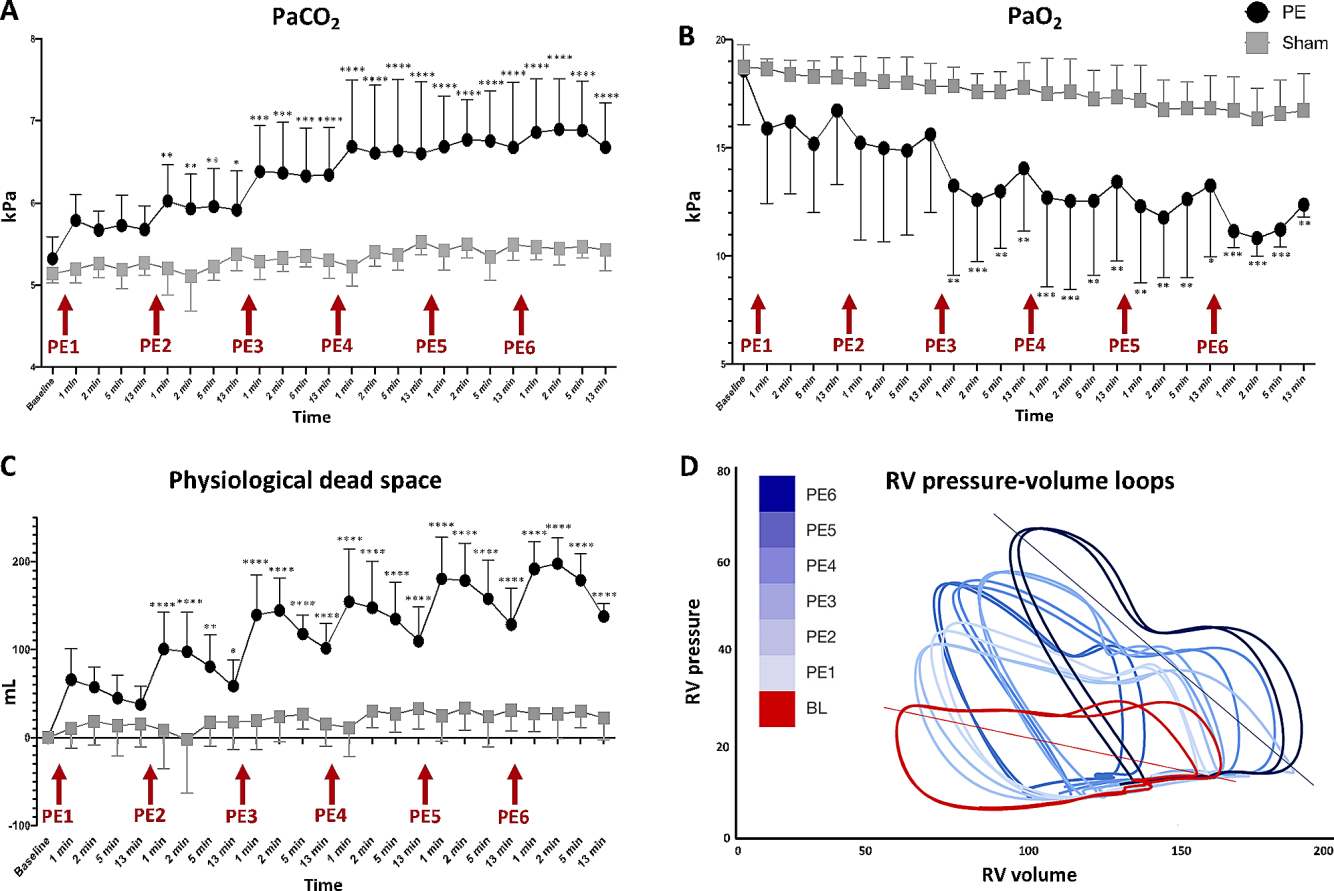
Ventilatory and pressure-volume loop responses to consecutive acute pulmonary embolism Consecutive, acute pulmonary embolism caused changes in arterial partial pressure of carbon dioxide PaCO_2_, **A**) and oxygen (PaO_2_, **B**) as well as physiological dead space (**C**), all in stepwise manners. (**D**) shows representative pressure-volume loops from the right ventricle (RV) from baseline (BL) and after consecutive pulmonary embolism (PE). Note how both volume (dilatation) and pressure (afterload) increases with repeated PE. Furthermore, the shape of the loop changes with an increasingly evident end-systolic notch on the loop representing increased afterload and early wave reflection. At baseline and last loop (PE6), the arterial elastance (Ea) lines are drawn with the increased slope representing increased afterload

Consecutive PEs led to a stepwise increase in PaCO_2_ (Fig. 4A) and similar PaO_2_ decrease (Fig. 4B), while pulmonary shunt and physiological dead space increased consistently from baseline (Fig. 4C).

Clot mass correlated to ventilatory function in PE animals, with some correlations observed in sham animals, albeit with different correlation coefficients (*p* < 0.0001 for all). See Table 1.

### Systemic circulation

Consecutive PE induced increases in heart rate (HR) and central venous pressure (CVP) compared to baseline (Supplementary Fig. 1A-B). Systolic blood pressure transiently dropped with each PE, but not statistically significantly (Supplementary Fig. 1C). Cardiac output (CO) showed compensatory increases initially, followed by a decrease (not statistically different in overall ANOVA analysis, Supplementary Fig. 1D).

### Left ventricle

LV PV data were similar between sham and PE animals at baseline. Inductions of PE caused a tendency to underfilled LV with reduced LV pressures and volumes, most evident for LV end-diastolic volume and LV stroke work (Supplementary Fig. 2), but this was not statistically different (data not shown).

## Discussion

In this study we evaluated immediate and early cardiopulmonary responses to successive PEs revealing time-dependent variable cardiopulmonary responses. We showed that pulmonary arterial (PA) pressure increased as an early response compared to RV afterload and function, which were affected at a later point.

Our observations of RV dilatation, afterload increase and increased pressure from successive PE episodes are all evident in representative RV pressure-volume loops (Fig. 4D).

### RV afterload and pulmonary vasculature

We confirmed an increase in mPAP from acute PE [9–11, 13]. However, our observations underscored acute PE being a highly dynamic condition as the largest mPAP increase was induced by the very first PE with smaller and stepwise increments in mPAP by the following PEs. Of particular interest, the PA pressure almost normalized within 15 min (i.e. before the second PE, see Fig. 2A). This may explain the clinical experience of patients reporting severe symptoms from the index event while being only slightly affected at the time of hospital admission. Other animal models have similarly shown excessive but transient mPAP increases with a following stabilization period [17, 18]. The prominent PA pressure response seen with the first of a series of consecutive emboli could possibly be related to an initial strong vasoconstrictor response that are weaker for the following emboli. However, we know that there is a substantial contribution of pulmonary vasoconstriction in acute PE that persists for hours, as we have previously shown prominent effects of pulmonary vasodilatory agents in this model [19, 20].

We observed that PVR and PA elastance did not immediately surge despite early increases in mPAP. Initial compensatory mechanisms like CO elevation or recruitment of non-embolized lung regions might underlie this delay, which is supported by the observation of an immediate increase in PVR/SVR ratio where CO is omitted from the equation.

The correlation between clot burden and pulmonary pressure as seen in this experimental study has also been observed in clinical studies [21–23]. Unfortunately, clot burden scores are poor predictors of clinical outcomes [24, 25], whereas pulmonary trunk dilatation, as a marker of increased pulmonary pressure, has been associated with adverse outcome in acute PE [26, 27].

### Right ventricular function

RV adaptation to increased afterload involves complex changes including dilatation and increased contractility (Anrep effect). We observed RV dilatation comparable to other animal models of acute PE [11, 28]. The dilatation manifested early and plateaued at higher clot burdens, possibly due to pericardial constraints in our closed-chest approach. Secondly, we noticed a decrease in RV coupling (Ees/Ea) from the first PE which diverged from previous studies showing stepwise increases in RV Ea initially followed by decreased contractility and RV uncoupling at higher clot burdens [9, 11]. Differences in PE models and evaluation methods may explain this discrepancy.

RV dysfunction with reduced EF became apparent only when compensatory mechanisms were exhausted, aligning with increased Ea and PVR. Our observation of preserved CO combined with reduced RV EF confirms previous findings [17]. Compromised RV function but maintain systemic circulation mimics the intermediate-risk group according to ESC risk stratification [1].

In a clinical comparison, studies exhibit inconsistent associations between clot burden and RV dysfunction [29–31]. Clot burden association may be most pronounced in previously healthy individuals (comparable to our animals) where the compensatory mechanisms are larger [23, 32].

### Ventilatory function

Our observations align with some previous studies demonstrating stepwise changes in pulmonary variables during successive PEs [11, 16], emphasizing clot burden’s direct relationship with ventilation-perfusion mismatch. Our finding of hypercapnia is likely due to the animals being anesthetized and ventilated with a fixed respiratory frequency opposite to awake PE patients with tachypnea and hypocapnia [33].

### Limitations

Despite the strengths of our animal model mirroring human physiology [34], caution is warranted in translating our findings to clinical settings. Though patients may also experience successive PEs, it will occur less strictly and predictable than in our study design. The add-on cardiopulmonary responses from one PE to the next may therefore differ, also due to potential comorbidity of real-world patients. Secondly, while our model of PE relies on autologous blood, we acknowledge that our thrombi are quite fresh and uniform compared to a more heterogeneous picture in clinical settings. Thirdly, mechanical ventilation with a fixed respiratory rate affects the blood gasses.

## Conclusion

In an experimental model of consecutive PE, we showed an immediate and pronounced increase in PA pressure in response to the first PE, whereas consistent increase in RV afterload and onset of RV dysfunction ensued later after the third PE. Ventilatory variables were more directly associated with clot burden. The results emphasize the importance of hemodynamic evaluation in acute PE regardless of clot burden as well as the dynamics of acute PE where immediate cardiopulmonary changes may occur.

### Electronic supplementary material

Below is the link to the electronic supplementary material.

Supplementary Material 1

Supplementary Material 2

## Data Availability

The datasets used and/or analysed during the current study are available from the corresponding author on reasonable request.

## Abbreviations

3R: refine replace reduce
ANOVA: Analysis of variance
ARRIVE: Animal ResearchReporting of In Vivo Experiments
CO: cardiac output
CVP: central venous pressure
Ea: arterial elastance
EDV: end-diastolic volume
EF: ejection fraction
Ees: end-systolic elastance
ESP: end-systolic pressure
ESV: end-systolic volume
EtCO< Subscript>2</Subscript>: end-tidal carbon dioxide
HR: heart rate
IVC: inferior vena cava
mPAP: mean pulmonary arterial pressure
PA: pulmonary arterial
PaCO< Subscript>2</Subscript>: partial pressure of arterial carbon dioxide
PaO< Subscript>2</Subscript>: partial pressure of arterial oxygen
PE: pulmonary embolism
PV: pressure-volume
PvCO< Subscript>2</Subscript>: partial pressure of venous carbon dioxide
PvO< Subscript>2</Subscript>: partial pressure of venous oxygen
PVR: pulmonary vascular resistance
RV: right ventricle/ventricular
SV: stroke volume
SVR: systemic vascular resistance
