# Supplementary material for: Immediate cardiopulmonary responses to consecutive pulmonary embolism: a randomized, controlled, experimental study
Source: BMC Pulm Med. 2024 May 14;24:233. doi: 10.1186/s12890-024-03006-9 (PMC11093735; doi:10.1186/s12890-024-03006-9)
Supplement: Supplementary file 1 — Supplementary Material 1 [file 12890_2024_3006_MOESM1_ESM.pdf]

Supplementary figure 1:

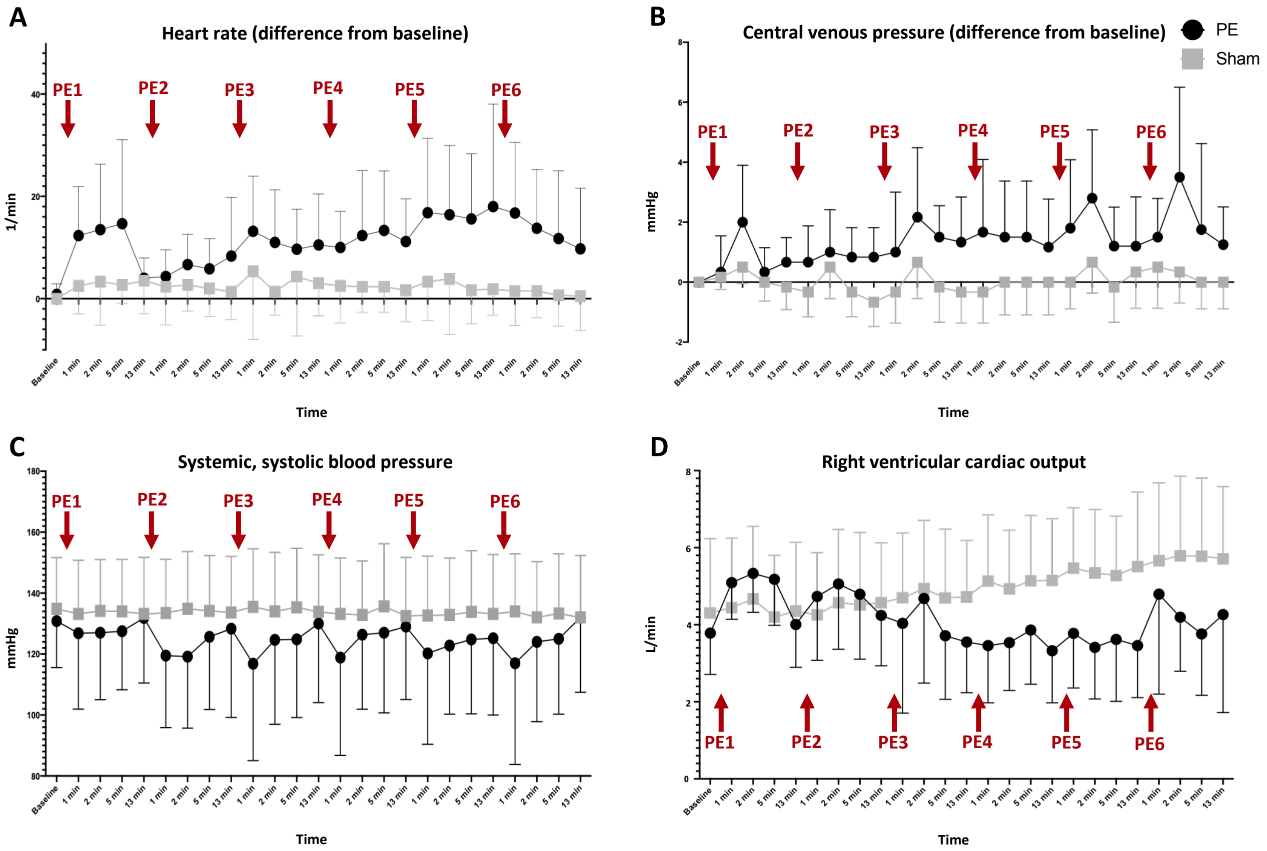

Title: Systemic variables in consecutive pulmonary embolism

Caption: Consecutive, acute pulmonary embolisms increased heart rate (A), mostly at the first embolism, and central venous pressure (B). Systolic, systemic blood pressure dropped transiently at every embolism induced (C). The cardiac output (D) was initially compensated by increased heart rate and stroke volume, but low output remained following the third pulmonary embolism. Please compare the latter to Figure 2C on right ventricular stroke volume.
