# Supplementary material for: Immediate cardiopulmonary responses to consecutive pulmonary embolism: a randomized, controlled, experimental study
Source: BMC Pulm Med. 2024 May 14;24:233. doi: 10.1186/s12890-024-03006-9 (PMC11093735; doi:10.1186/s12890-024-03006-9)
Supplement: Supplementary file 2 — Supplementary Material 2 [file 12890_2024_3006_MOESM2_ESM.pdf]

Supplementary figure 2:

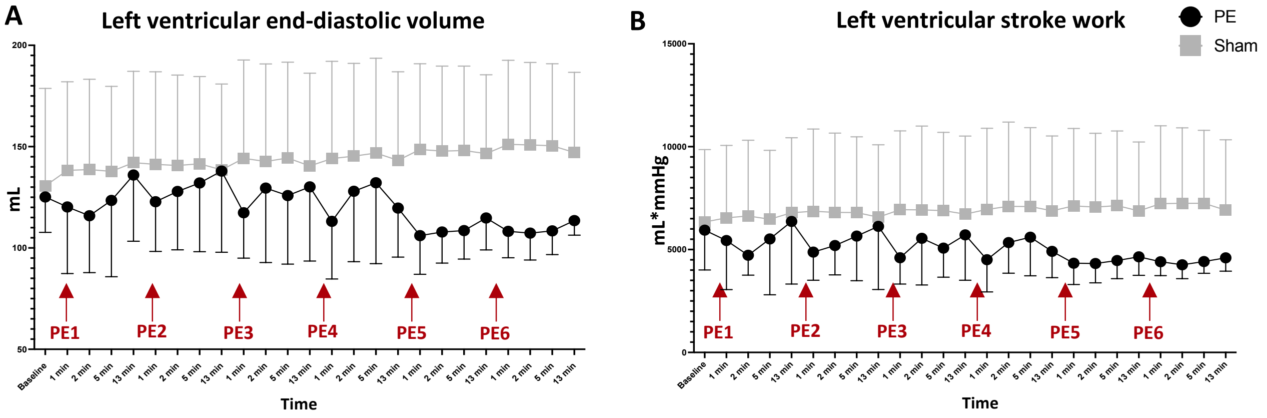

Title: Left ventricular pressure-volume data in consecutive pulmonary embolism

Caption: Consecutive, acute pulmonary embolisms caused decrease in left ventricular end-diastolic volume (A) as the two cardiac ventricles are connected in both serial and parallel. Please compare the reductions in left ventricular volume with the increase in right ventricular volume (i.e. dilatation, Figure 3B). B: Similarly, left ventricular stroke work (area of the pressure-volume loop, i.e. combination of both ventricular pressure and volume) was reduced after each consecutive embolism. None of the left ventricular pressure-volume data were statistically different.
